# Publisher Correction: COVID-19 in Europe: from outbreak to vaccination

**DOI:** 10.1186/s12889-022-14814-1

**Published:** 2022-12-23

**Authors:** Paula Vicente, Abdul Suleman

**Affiliations:** Instituto Universitário de Lisboa (ISCTE-IUL), Business Research Unit (BRU-IUL), Lisboa, Portugal

**Publisher Correction: BMC Public Health 22, 2245 (2022)**

**https://doi.org/10.1186/s12889-022-14454-5**

The original publication of this article [1] contained overlapping text on page 11. This error has been corrected. The publisher apologizes for the inconvenience caused to readers & authors.
